# Comparison of Two Routes of Administration of Dexamethasone to Reduce the Postoperative Sequelae After Third Molar Surgery: A Systematic Review and Meta-Analysis

**DOI:** 10.2174/1874210601812010181

**Published:** 2018-02-28

**Authors:** Giuseppe Troiano, Luigi Laino, Marco Cicciù, Gabriele Cervino, Luca Fiorillo, Cesare D’amico, Khrystyna Zhurakivska, Lorenzo Lo Muzio

**Affiliations:** 1 Department of Clinical and Experimental Medicine, Foggia University, Foggia, Italy; 2 Department of Biomedical, Dental Science and Morphological and Functional Images, Dental School, University of Messina, Italy

**Keywords:** Third molar, Wisdom tooth, Oral surgery, Corticosteroids, Postoperative pain, Edema, Trismus

## Abstract

The aim of this systematic review and meta-analysis was to compare the clinical efficacy of two routes of dexamethasone administration in reducing the postoperative sequelae after third molar extraction. Electronic databases (PUBMED, SCOPUS and EBSCO library) were screened in order to find both randomized and non-randomized clinical trials that directly compare the submucosal intraoral or the intramuscular extraoral administration of dexamethasone. No restriction about year of publication was imposed. About 340 titles and abstracts were screened independently by two authors. Of these [340 titles], only 4 randomized clinical trials met the inclusion criteria and were included in the meta-analysis. No statistical differences in postoperative pain, swelling and trismus were recorded comparing the intraoral submucosal and the extraoral intramuscular injection of dexamethasone in an extra-oral site.

## 1 INTRODUCTION

### 1.1 Rationale

The extraction of third molar is one of the most common procedures carried out by oral and maxillofacial surgeons in clinical practice [1, 2]. Due to the injury of surrounding tissues, such interventions are often associated with postoperative sequelae like pain, edema and trismus that strongly influence the patient's morbidity [3]. These side effects are triggered by an inflammatory response that affects the area of intervention leading to vasodilation and arrival of strong pro-inflammatory mediators [3, 4].

A wide array of drugs has been used with the aim to prevent the postoperative inflammation [4]. Among these, corticosteroids are one of the most used classes of drugs due to their strong anti-inflammatory activity and relative safety in healthy patients [4]. Corticosteroids are able to reduce fluid transudation, inhibit vascular dilation and decrease cell updates through a reduction of chemotaxis of inflammatory cells and inhibition of the production of several inflammatory mediators [5, 6].

Different routes of corticosteroids administration have been tested, but there is no consensus about the best treatment approach because every administration method presents advantages and disadvantages [7, 8]. Intramuscular, submucosal, intravenous and intra-alveolar powders have all been demonstrated to reduce the postoperative sequelae after surgical extraction of mandibular third molars [9-11]. Major interest has been addressed to the possibility of reducing the postoperative sequelae with an intraoral submucosal injection compared to an extra-oral intramuscular administration, which is still considered the classical approach. The possibility to inject the drug in proximity of the surgical site, still in the presence of anesthetic effect, represents a promising option in order to decrease the discomfort of an injection in a different site just after the end of the surgical operation.

### 1.2 Objectives

The aim of this systematic review and meta-analysis was to compare the effects of dexamethasone on postoperative pain, swelling and trismus when administered submucosally close to the site of the extraction or intramuscularly in an extraoral site just after mandibular wisdom tooth removal. The null hypothesis of the study was that there is no difference between the two ways of administration in the reduction of the postoperative sequelae after third molar surgery.

## 2 MATERIALS AND METHODS

### 2.1 Protocol, Registration and Eligibility Criteria

The protocol of this systematic review has been developed according to the Cochrane Handbook [12]and the Preferred Reporting Items for Systematic reviews and Meta-Analysis (PRISMA) [13]. In addition, the systematic review has been registered on the PROSPERO database (registration number: CRD42016035221). Studies published in English language and fulfilling the following criteria were considered eligible for inclusion in this review:

1. Both randomized and non-randomized clinical trials directly comparing the submucosal and intramuscular injection of dexamethasone after third molar extraction.
2. Both studies with parallel- or split mouth- design.
3. No restriction about the number of arms present in the studies.
4. At least ten patients treated in each group.
5. No restriction about the year of publication.

### 2.2 Information Sources and Search Strategy

Studies were identified by searching the following electronic databases: PUBMED, EBSCO li- brary and SCOPUS. The research was carried out independently by two authors (GT and LL) in the period between the [first] and [eighth] of February 2016. [Combinations]of MeSH terms and free text word have been combined using Boolean operators. The following terms have been used:

Type of intervention: (“ dexamethasone ” [Mesh] OR “ corticosteroid” [free word])

AND

Disease and site concerned: (“ third molar ” [Mesh] OR “ wisdom tooth ” [free words] OR “third molars” [free words])

AND

Study design: (“randomized controlled study” [Mesh] OR “prospective study” [Mesh] OR “comparative study” [Mesh]).

A direct online research on the official sites of: Journal of Oral and Maxillofacial Surgery, International Journal of Oral & Maxillofacial Surgery, British journal of oral and maxillofacial surgery and Journal of Craniofacial surgery was also performed. In addition, a direct research on bibliographies of articles read full-text was carried out, in order to find other articles eligible for inclusion in this systematic review.

### 2.3 Study Selection, Data Collection Process and Data Items

The assessment of eligibility for inclusion was carried out independently by two reviewers (GT and MC). In the first round, the evaluation was performed reading the title and abstract of the studies, disagreements between reviewers was resolved through discussion. Articles who met the inclusion criteria were subsequently read full-text. In the second screen, the authors excluded studies which did not meet the criteria regarding: participants, intervention characteristics, comparisons, outcome measures and study design (PICOS). The data collection was performed trough an *ad hoc* extraction sheet. For each study, information was extracted according to PICOS [14]. The following data were extracted: number of patients and teeth extracted in the two groups, mean age of participants year of publication (Participants); dose of dexamethasone administered, schedules of post-surgical medications, classification of third molar impaction, design of the flap (Intervention Characteristics); methods for evaluation of pain, swelling and trismus (Comparisons); outcomes measurement at 1, 3 and 7 days for pain swelling and trismus (Outcomes) and the design of the studies including the number of arms (Study design).

### 2.4 Risk of Bias in Individual Studies

The assessment of risk of bias in individual studies was evaluated using the Cochrane Collaboration tool for randomized clinical trials [15]. The analysis of each study was based on six criteria: appropriate sequence generation, concealment of the allocation sequence, blinding of participants, incomplete outcome data, selective outcome reporting and other sources of bias.

### 2.5 Planned Methods of Analysis and Risk of Bias Across Studies

Data for primary (Postoperative Pain) and secondary (Swelling and Trismus) outcomes were interpolated to evaluate the Mean difference or the Standardized Mean Difference of the effects estimated. The Higgins Index and the Chi-squared test were used to assess the heterogeneity and classified as follow: *I^2^* <30% low heterogeneity, *I^2^ =*30-60% medium heterogeneity, *I^2^* >60% high heterogeneity [16]. For the primary outcome, also, a graphical evaluation, building a funnel plot, was performed for assessment of heterogeneity [17]. The statistical analysis was carried out entering: mean, standard deviation and sample size, into Review Manager version 5.2.8 (Cochrane Collaboration, Copenhagen, Denmark; 2014). Inverse of variance test was used at fixed or random effects models on the basis of studies heterogeneity. Whether *I^2^* was lower than 60% a fixed effect model was used, while if *I^2^* was higher than 60% data were pooled using a random effect model.

## 3 RESULTS

### 3.1 Study Selection

**Fig. (1):**
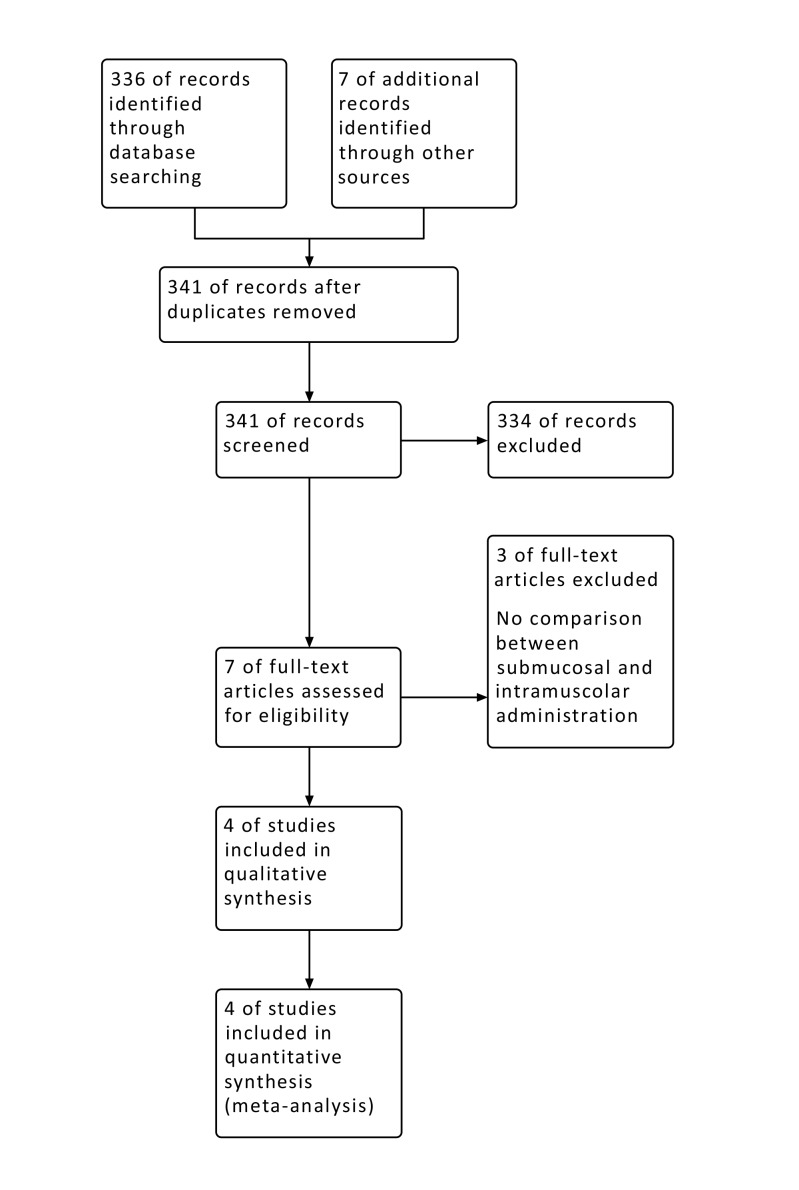
Flow chart graph of review process.

A total of 341 records were screened from databases on title and abstract. Only 11 out of these studies met the inclusion criteria and were considered eligible for the full-text examination (Fig. **1**). At the end of full-text examination, only 4 papers met the inclusion criteria and were included in the meta- analysis [18-21]. Three articles were excluded because they did not report about a direct comparison between intramuscular and submucosal administration of dexamethasone [7, 8, 11].

### 3.2 Study Characteristics

The four studies included in the meta-analysis were all prospective randomized clinical trials; the characteristics of the studies included are summarized in Table **1**. In all the studies, the dose of dexamethasone administered was of 4 mg. The number of participants in the studies ranged from 10 to 12 for each group. The mean age of participants ranged from (24.1) to 27 years, all the studies had a parallel design with the number of arms ranging from 3 to 6. The Pell and Gregory classification [22]was used to categorize the type of impaction in all the included studies. For the access to the impacted tooth a triangular flap was used in three studies, while a modified ward's flap in the remaining one [21]. All the studies have assessed the postoperative pain with a VAS scale and the trismus as the difference in maximum mouth open before and after the surgery. Different methods were used to assess the edema in the included studies: two studies [18, 19] measured two distances (Tragus-midline and Gonion-lateral canthus), while the other two studies used one [21] (Tragus-menton) or three [23](Tragus-midline, Gonion-lateral canthus and Tragus-canthus of the mouth) measurements (Table **2**).

### 3.3 Risk of Bias Within Studies

The risk of bias assessment revealed that no studies were free of bias in Table **3**. Regarding the quality of randomization, it was adequate in three studies who reported about the method of randomization [18, 19, 23], while another study did not report type of sequence generation [21]. The blinding of participants was present only in one study [21], while was absent in the remaining three [18, 19, 23]. Incomplete data outcomes and selective reporting bias have not been found. Another source of bias found for three studies relating to the post-surgical medication reported that the use of Tramadol was administered as needed. This administration, in the opinion of the authors, could cause errors because a different amount of analgesic assumed by patients may influence the postoperative pain.

### 3.4 Postoperative Pain

**Fig. (2):**
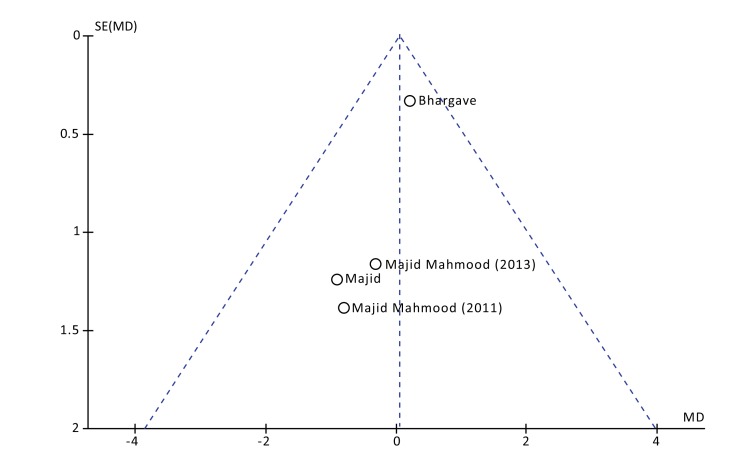
Funnel plot for the outcome pain of included studies.

**Fig. (3):**
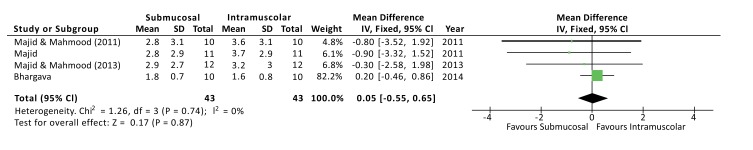
Forest plot at fixed effect model for the outcome pain, one day after surgery.

The postoperative pain was evaluated at 1, 3 and 7 days after surgery. No statistical differences were found at all the time points. Both the Higgins Index (I2 = 0%) and the funnel plot revealed the absence of heterogeneity (Fig. **2**). At 1 day after surgery the Mean Difference (D) between the two methods was 0.05 with a 95% Confidence Interval (CI) from -0,55 to 0,65 and a *p*-value (*P*) for overall effects of 0,87 (Fig. **3**). At 3 days from surgery, the comparison revealed a MD = 0,12 (95% CI -0,51 to 0,75) with *P* = 0,71. While, at 7 days from surgery, there was a MD = 0,07 (95% CI -0,19 to 0,33) and *P* = 0,58.

### 3.5 Swelling

The severity of swelling after the surgery was evaluated at 1, 3 and 7 days, and no differences were found at all the time points. Although the outcome swelling (or oedema) has been evaluated, in the studies, with different methods of measurement, a standardized mean difference (SMD) was used to interpolate the effects size [3]. At 1 day from surgery the SMD between the two methods was 0.03 with a 95% CI from -0,39 to 0,45 and a *p*-value for overall effects of 0,88 Fig. (**4**). At 3 days from surgery the comparison revealed a SMD = 0,31 (95% CI -0,12 to 0,74) with *P* = 0,15, while, at 7 days from surgery there was a SMD = -0,26 (95% CI -0,69 to 0,16) and *P* = 0,22. No sources of heterogeneity (*I2* = 0%) have been found at all the time points analyzed.

### 3.6 Trismus

No differences have been found at all the time points regarding the reduction in the maximum mouth opening at 1, 3 and 7 days. No sources of heterogeneity (I2 = 0%) have been found at all the time points analyzed. At 1 day from surgery the MD between the two methods was 0,27 with a 95% CI from -0,57 to 1,12 and a *p*-value for overall effects of 0,53. At 3 days from surgery the comparison revealed a MD = -0,05 (95% CI -1,25 to 1,15) with *P* = 0,15, while, at 7 days from surgery there was a MD = 0,05 (95% CI -0,87 to 0,96) and *P* = 0,92.

## 4 DISCUSSION

Overcoming the selection process, 4 randomized clinical trials (RCTs) met the inclusion criteria and were included in the meta-analysis. RCTs represent the highest level of scientific evidence, but all the 4 articles included presented some sources of bias. In the evaluation of risk of bias within studies, none of the studies met all the characteristics in terms of: sequence generation, allocation concealment, blinding of participants, incomplete outcomes, selective reporting bias and other sources of bias. Relating to the risk of bias across studies, the heterogeneity evaluated with the Higgins Index and funnel plots was very low, demonstrating almost the absence of bias. In the evaluation of risk of bias, another source of bias has been found in three studies regarding the post-operative administration of analgesic. In fact, if the analgesia is administered “as needed” this could lead to a different intake by patients, and influence the postoperative pain. Academic bias could also be present, because three studies [18, 19, 23] came from the same University, but it is minimized by the inclusion of the study of Bhargava [23]. In all the included studies, dexamethasone was administered immediately after surgery at a dose of 4 mg. All the studies had a parallel design with a number of arms ranking from 3 to 6, and no split-mouth studies were found in the literature regarding the topic in question. The severity of impaction has been classified according to the Classification of Pell and Gregory in all the included studies, although the reliability of this classification has been recently questioned [24]. In all the three studies [18, 19, 23], a triangular flap was used to access the impacted tooth, while in another study the access was performed with a modified ward's flap [23]. Regarding the method of measurement all the studies used a VAS scale for evaluating the postoperative pain and the distance in maximum mouth opening for the evaluation of trismus. By contrast, heterogeneity was detected regarding the measurement of swelling. Since different parameters were taken into consideration for the measurement of such outcome; a SMD was calculated only for these parameters. Looking at results, no differences have been found, in terms of pain, swelling and trismus, regarding the administration of dexamethasone for submucosal or intramuscular administrations. Because the heterogeneity calculated was very low for all the outcomes, at all the time points, a fixed effect model was used to evaluate the size of effects in all the parameters. In a recent meta-analysis of randomized clinical trials (RCTs), the clinical efficacy of submucosal injection of dexamethasone has demonstrated to reduce the postoperative edema and pain, while no difference has been demonstrated for the reduction of trismus [25]. However, no meta-analysis of RCTs has been found regarding the intramuscular administration.

## CONCLUSION

No statistical differences have been found in relation to postoperative pain, swelling and trismus, regarding the submucosal or intramuscular injection of dexamethasone. The choice between the two routes of administration should not be linked to the clinical efficacy in reducing the postoperative sequelae, but in relation to the discomfort during administration which should be assessed in future studies.

## Figures and Tables

**Fig. (4):**
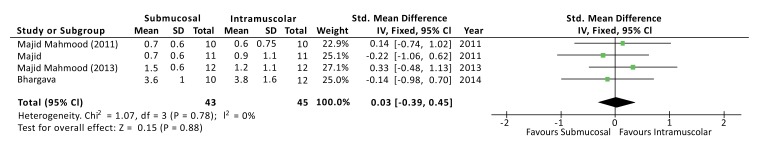
Forest plot at fixed effect model for the outcome swelling one day after surgery.

**Table 1:** Characteristics of studies included in meta-analysis.

**Author**	**Year**	**Dose of Dexamethasone**	**Patients**		**Mean Age**	**Design of the Study**	**Number of teeth Extracted**		**Classification of Impaction**	**Design of Flap**	**Postoperative Administration**
–	–	–	Submucosal	Intramuscolar	–	–	Submucosal	Intramuscolar	–	–	–
**Majid & Mahmood**	2011	4 mg	10	10	27	Parallel (3 groups)	10	10	Class II and III A, B, C Pell and Gregory	Triangular mucoperiosteal	Amoxicillina 500 mg every 8 h and Tradamol Tablets 50 mg
**Majid**	2011	4 mg	11	11	26,9	Parallel (3 groups)	11	11	Class II and III B, C Pell and Gregory	Triangular mucoperiosteal	Amoxicillina 500 mg every 8 h and Tradamol Tablets 50 mg arequrequiredmg as required
**Majid & Mahmood**	2013	4 mg	12	12	25,6	Parallel (6 groups)	12	12	Class II and III A, B, C Pell and Gregory	Triangular mucoperiosteal	Amoxicillina 500 mg every 8h and Tradamol Tablets 50 mg requiredmg
**Bhargava **	2014	4 mg	10	10	24,1	Parallel (6 groups)	10	10	Class II position B Pell and Gregory	Modified ward's mucoperiosteal	Amoxicillina 500 mg every 8 h and Paracetamol 650 mg for 5 days

**Table 2:** Data extracted for outcomes: pain, swelling and trismus.

**Author**	**Method Evaluation of Pain**			**Postoperative Pain DAY 1**							**Postoperative Pain DAY 3**						**Postoperative Pain DAY 7**			
**–**	–			TEST (Submucosal)			CONTROL (Intra-Muscolar)				TEST (Submucosal)			CONTROL (Intra-Muscolar)			TEST (Submucosal)			CONTROL (Intra-Muscolar)
**Majid & Mahmood** **(2011)** ****	VAS 100mm			2,8 ± 3,1			3,6 ± 3,1				1,5 ± 2,4			1,1 ± 2,1			0,2 ± 0,6			0,2 ± 0,6
**Majid** **(2011)**	VAS 100mm			2,8 ± 2,9			3,7 ± 2,9				1,5 ± 2,3			1,2 ± 2			0,2 ± 0,6			0,2 ± 0,6
**Majid & Mahmood** **(2013)** ****	VAS 10 cm			2,9 ± 2,7			3,2 ± 3				1,4 ± 2,3			1,1 ± 2			0,2 ± 0,6			0,2 ± 0,6
**Bhargava (2014)**	VAS Ten points			1,8 ± 0,7			1,6 ± 0,8				1,2 ± 0,9			1,2 ± 0,9			1,0 ± 0,9			0,6 ± 0,6
**Author**	**Method Evaluation of Swelling**		**Swelling DAY 1**						**Swelling DAY 3**						**Swelling DAY 7**					
**–**	–		TEST (Submucosal)			CONTROL (Intra-muscolar)				TEST (Submucosal)			CONTROL (Intra-muscolar)		TEST (Submucosal)			CONTROL (Intra-muscolar)		
**Majid & Mahmood** **(2011)** ****	Tragus-midline Gonion-lateral canthus		0,7 ± 0,6			0,6 ± 0,75				0,5 ± 0,6			0,3 ± 0,42		0,06 ± 0,2			0,05 ± 0,15		
**Majid** **(2011)**	Tragus-midline Gonion-lateral canthus		0,7 ± 0,6			0,9 ± 1,1				0,5 ± 0,5			0,5 ± 0,6		0,06 ± 0,1			0,1 ± 0,2		
**Majid & Mahmood** **(2013)** ****	Tragus-midline Gonion-lateral canthus Tragus-canthus of the mouth		1,5 ± 0,6			1,2 ± 1,1				1,2 ± 0,5			0,7 ± 0,6		0,1 ± 0,1			0,1 ± 0,2		
**Bhargava (2014)**	Tragus-menton		3,6 ± 1,0			3,8 ± 1,6				3,0 ± 1,0			3,0 ± 1,3		0,3 ± 0,4			0,4 ± 0,5		
**Author**	**Method evaluation of swelling**	**Swelling DAY 1**						**Swelling DAY 3**								**Swelling DAY 7**				
**–**	–	TEST (Submucosal)			CONTROL (Intra-muscolar)			TEST (Submucosal)				CONTROL (Intra-muscolar)				TEST (Submucosal)			CONTROL (Intra-muscolar)	
**Majid & Mahmood** **(2011)** ****	Maximal mouth opening	10,8 ± 9,4			14,5 ± 9,3			8,2 ± 8,8				10,4 ± 8,8				5,4 ± 7,9			5,1 ± 4,3	
**Majid** **(2011)**	Maximal mouth opening (difference post-pre)	11 ± 9			14 ± 9			8,5 ± 8,4				10 ± 8,4				5,5 ± 7,5			4,8 ± 4,2	
**Majid & Mahmood** **(2013)** ****	Maximal mouth opening	11,5 ± 9			14 ± 9			8,6 ± 8,4				10 ± 8,4				5,2 ± 7			4,8 ± 4,2	
**Bhargava (2014)**	Maximal mouth opening (difference post-pre)	2,3 ± 1,2			1,9 ± 0,7			2,0 ± 1,25				1,9 ± 1,6				0,9 ± 1,1			0,9 ± 1,1	

**Table 3:** Results about risk of bias in the included studies.

Study	Sequence Generation	Allocation Concealment	Blinding	Incomplete Outcomes Data	Selective Reporting Bias	Other Sources of Bias
Majid (2011)	YES	YES	NO	NO	NO	YES
Majid and Mahmood (2011)	YES	YES	NO	NO	NO	YES
Majid and Mahmood (2013)	YES	YES	NO	NO	NO	YES
Barghava (2014)	UNCLEAR	UNCLEAR	YES	NO	NO	NO
